# Evaluating physiological responses of plants to salinity stress

**DOI:** 10.1093/aob/mcw191

**Published:** 2016-09-05

**Authors:** S. Negrão, S. M. Schmöckel, M. Tester

**Affiliations:** King Abdullah University of Science and Technology (KAUST), Division of Biological and Environmental Sciences and Engineering, Thuwal, 23955-6900, Saudi Arabia

**Keywords:** Assessing salinity tolerance, salt-imposition systems, quantifying physiological traits, analysing salinity data, tolerance indices, salt stress phenotyping, osmotic stress

## Abstract

**Background** Because soil salinity is a major abiotic constraint affecting crop yield, much research has been conducted to develop plants with improved salinity tolerance. Salinity stress impacts many aspects of a plant’s physiology, making it difficult to study *in toto*. Instead, it is more tractable to dissect the plant’s response into traits that are hypothesized to be involved in the overall tolerance of the plant to salinity.

**Scope and conclusions** We discuss how to quantify the impact of salinity on different traits, such as relative growth rate, water relations, transpiration, transpiration use efficiency, ionic relations, photosynthesis, senescence, yield and yield components. We also suggest some guidelines to assist with the selection of appropriate experimental systems, imposition of salinity stress, and obtaining and analysing relevant physiological data using appropriate indices. We illustrate how these indices can be used to identify relationships amongst the proposed traits to identify which traits are the most important contributors to salinity tolerance. Salinity tolerance is complex and involves many genes, but progress has been made in studying the mechanisms underlying a plant’s response to salinity. Nevertheless, several previous studies on salinity tolerance could have benefited from improved experimental design. We hope that this paper will provide pertinent information to researchers on performing proficient assays and interpreting results from salinity tolerance experiments.

## INTRODUCTION

Soil salinity is a global problem that affects approx. 20 % of irrigated land and reduces crop yields significantly (Qadir *et al.*, 2014). The physiological responses of a plant to salinity are often complex and multi-faceted, which makes experiments difficult to design and interpret. Current plant physiology has advanced, given the development of so-called ‘omics-driven’ research. Physiological measurements have been revolutionized by new technologies, such as high-throughput phenotyping, bioinformatics and novel analytical methods that have enabled fields such as metabolomics to emerge. At a basic level, the response of plants to salinity can be described in two main phases: the shoot ion-independent response occurs first, within minutes to days, and is thought to be related to Na^+^ sensing and signalling (Gilroy *et al.*, 2014; Roy *et al.*, 2014). In this first phase, effects of salinity on water relations can be important, causing stomatal closure and the inhibition of leaf expansion (Munns and Termaat, 1986). The second phase, the ion-dependent response to salinity, develops over a longer period (days to weeks) and involves the build-up of ions in the shoot to toxic concentrations, particularly in old leaves, causing premature senescence of leaves and ultimately reduced yield or even plant death (Munns and Tester, 2008).

Three main salinity tolerance mechanisms have been proposed by Munns and Tester (2008): ion exclusion – the net exclusion of toxic ions from the shoot; tissue tolerance – the compartmentalization of toxic ions into specific tissues, cells and subcellular organelles; and shoot ion-independent tolerance – the maintenance of growth and water uptake independent of the extent of Na^+^ accumulation in the shoot. Other physiological components are also likely to contribute to salinity tolerance, such as the maintenance of plant water status, transpiration (T) and transpiration use efficiency (TUE) (Harris *et al.*, 2010; This *et al.*, 2010; Barbieri *et al.*, 2012); leaf area (Maggio *et al.*, 2007); seed germination (Foolad and Lin, 1997); production of antioxidants (Ashraf, 2009); early seedling growth (Kingsbury and Epstein, 1984); and harvest index (HI) (Gholizadeh *et al.*, 2014). Very little is known about these physiological components, so understanding the effects of salinity on these processes needs further investigation. In addition, numerous factors can influence plants’ responses to salinity due to the complex nature of salinity tolerance. For example, the beneficial effect of calcium application to plants exposed to high levels of Na^+^ was reported back in 1902 by Kearney and Cameron (as reviewed by Lahaye and Epstein, 1971). Since then, the interaction between Na^+^ and Ca^2+^ has been extensively studied (as reviewed by Cramer, 2002), and nowadays several salinity experiments use Ca^2+^ supplemented to the medium to maintain Ca^2+^ activity (for further details see Supporting Methodologies, section 1).

In this review, we describe techniques that measure the impact of salinity on several physiological traits, such as growth, water relations, ion homeostasis, photosynthesis, yield components and senescence. It often can be difficult to identify which traits are the most important ones contributing to salinity tolerance in the given plant system. To ease this difficulty, we suggest the generation of graphs that show correlations between the proposed traits (e.g. leaf Na content) and a measure of salinity tolerance (e.g. salt tolerance index). Such correlations help to establish whether the measured traits are associated with each other (noting the limitation that a correlation cannot give definitive information on cause-and-effect relationships); the correlation coefficient can give an indication of which traits are the most important contributors to salinity tolerance (for the analysed plant in the analysed environment). Numerous studies have used two contrasting genotypes to characterize their salinity response at the transcriptional (e.g. Ouyang *et al.*, 2007; Beritognolo *et al.*, 2011), proteomic (e.g. Ma *et al.*, 2012; Cui *et al.*, 2015) or metabolomic (e.g. Widodo *et al.*, 2009; Zhao *et al.*, 2014) levels, but limited arguments advocate the reasoning of selecting such genotypes. When a selection of few contrasting genotypes is necessary, one should take into account the potential variability of the trait under study within the population and, if available, also consider genotypic information. Although the use of contrasting genotypes in such analyses is valid, we consider that this narrow selection is not representative of a species’ performance under salinity stress. Thus, we strongly encourage broadening these types of analyses by using several genotypes before speculating about a species’ performance.

We also suggest some guidelines for designing experiments and analysing data related to salinity tolerance, with the aim of facilitating the gathering and interpretation of accurate and useful physiological data. We have included Supporting Methodologies Section 1 in an attempt to facilitate the use of good quality experimental procedures that are crucial to the success of salinity studies. Key aspects include the experimental system (e.g. agar plates, hydroponics, soil-filled pots or the field), the extent of the salinity stress (levels of salt stress, timing of salt application and duration of treatment) and the biological system (species and genotype). In Supporting Methodologies Section 2, we provide further details on the indices explained in this review and how they can be derived from physiological measurements. Readers are also referred to excellent online resources, such as Prometheus Wiki (e.g. http://prometheuswiki.publish.csiro.au/tiki-index.php?page=Salinity) and the PlantStress website (http://www.plantstress.com/methods/index.asp).

The aim of providing extensive details in the Supporting Methodologies is to help new researchers as they begin to work in this field. That said, we also believe the extensive details are necessary because many papers are still being published with insufficient attention to important aspects of the experiments. Previously, Flowers (2004) examined in detail several papers that claimed significant effects of transgenic events on salinity tolerance. However, these papers were found to have provided insufficient evidence for the claims made for a range of reasons. Moreover, Claeys *et al.* (2014) also concluded that most of the published studies use very high levels of NaCl, and that the recorded phenotypes, which include expression data, are associated with severe stress responses. We hope that researchers new to this field can draw on some of the technical points raised in this paper and that new and experienced researchers alike will base their work on rigorous experimental methodologies such as those described in the Supporting Methodologies.

### Quantifying the effects of salinity on plant growth: destructive and non-destructive approaches

**Fig. 1.**
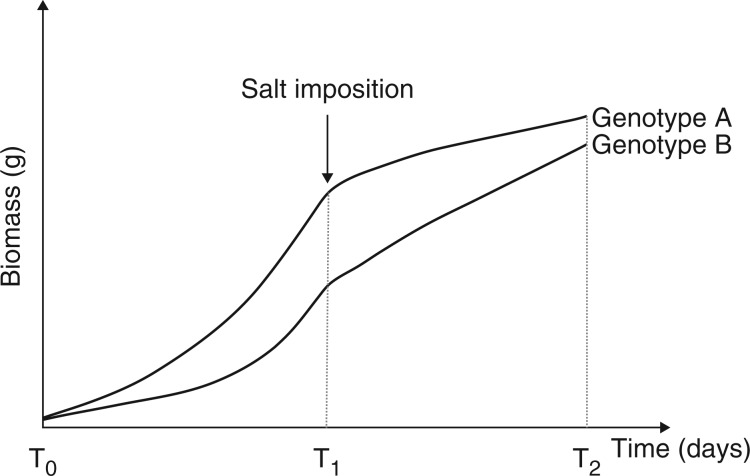
Salinity tolerance should be calculated by measuring the effects of salinity on plant growth during the time of stress imposition and not during the lifetime of the plant. Growth of two hypothetical genotypes is shown, before (T_0_ to T_1_) and after (T_1_ to T_2_) imposition of salinity stress. Genotype A grows faster than Genotype B under control conditions, but its growth is inhibited more by salinity. If growth were measured by biomass increase from T_0_ to T_2_, Genotype A would appear to be more salt tolerant. However, if growth were measured only from T_1_ to T_2_, then Genotype B would appear to be more salt tolerant.

In an experimental setting, one of the first observable responses after salinity imposition is a reduction in shoot growth (Fig. 1, Supplementary data Movie S1). To describe this reduction in plant growth, two distinct approaches can be used: a destructive harvest, or a non-destructive approach using, for example, digital imaging.

A destructive harvest involves separation of plants into parts, such as shoot from root, or into more parts, such as blade, petiole (or sheath), stem and root. Fresh mass and dry mass are then recorded; and other measurements can also be made, such as root length, plant height and leaf area. For detailed information about the key aspects to consider when designing such an experiment (e.g. selection of appropriate controls, stress imposition and the experimental system), see Supporting Methodologies Section 1. Although this destructive approach requires no specialized, expensive equipment, it often involves a substantial amount of manual handling, which, when combined with available space for growing plants, limits the number of time points for sampling. The relative decrease in plant biomass (RDPB) indicates the reduction in growth by comparing the total biomass of stressed and control plants at the end of the experiment [Supporting Methodologies Section 2, eqn (a)]. At a minimum, plants should be harvested at the time of the start of the salt stress and at the end of the period of interest, allowing the reduction in growth by salinity to be observed across the stress interval and not as an integral of the growth both before and after the salt stress. This is particularly important when comparing lines that have different rates of growth under control conditions and thus are likely to be different sizes at the beginning of the stress period. The stress interval can be defined as starting at the time of stress imposition (T_1_) until the endpoint of the experiment (T_2_, see Fig. 1). Measurements of the difference in growth reduction between control and stress treatments will be more accurate and usually greater when biomass increases are analysed only during the stress interval (T_1_ to T_2_).

It is important to mention that for plants that accumulate extremely high levels of salt in the shoot (e.g. halophytes such as *Salicornia*, *Suaeda*), the mass of the salt can become a significant fraction of the total mass. Hence, in these specific cases, we recommend the use of ash-free dry mass or ethanol-insoluble dry mass to quantify the reduction in growth – or at least to subtract the mass of NaCl from the total dry mass, given the amount of NaCl accumulated in the relevant plant part is known.

Another approach that should be considered is the use of dose–response curves, which can be applied to an individual or to several genotypes. In *Arabidopsis*, the evaluation of dose–response curves in salinity has shown that low concentrations of salt have little effect on growth, but at higher concentrations the relative growth rate quickly decreases as a quadratic function of the NaCl concentration (Claeys *et al.*, 2014). A meta-analysis study using response curves showed that leaf-related parameters (i.e. leaf-specific area and leaf dry mass per unit area) were significantly affected by salinity (Poorter *et al.*, 2009, 2010). Although time consuming to perform and analyse, dose–response curves may provide valuable insights into the genotypic and phenotypic differences in response to salinity. In the future, the use of big-data analysis and statistical methods may enable dose–response curves to be considered in associated studies.

The second approach for assessing plant growth in response to salinity uses image acquisition technology. In this non-destructive approach, images of plants are taken at defined time intervals and the biomass is deduced from pixel counts (Berger *et al.*, 2012). With automated, high-throughput phenotyping facilities, such as The Plant Accelerator in Adelaide, Australia, and in facilities hosted by other partners of the International Plant Phenotyping Network (http://www.plant-phenotyping.org/), it is possible to obtain a daily estimate of plant biomass from the start of an experiment, before salt imposition, through to the end. These systems are powerful because they provide high time and spatial resolution. Because of this high resolution, it is possible to develop more detailed models of growth (Ward *et al.*, 2015) and to estimate relative growth rates (RGRs) (Berger *et al.*, 2012), as has recently been achieved for rice (Hairmansis *et al.*, 2014; Campbell *et al.*, 2015). Importantly, these measurements also allow assessment of the shoot’s ion-independent component of salt toxicity, which involves the inhibition of shoot growth from the moment of salt imposition (Berger *et al.*, 2012), before salt has had time to accumulate in the shoot and significantly affect the shoot’s function [Supporting Methodologies Section 2, eqn (b)].

Image acquisition technologies are developing rapidly and a number of software tools are now freely available (http://www.plant-image-analysis.org/), enabling the efficient analysis of imaging data. To date, a plethora of imaging systems focus on shoot parameters (as recently reviewed by Fahlgren *et al.*, 2015). These systems appear to be particularly robust and reliable to quantify shoot growth under controlled environments. Few systems to date have evaluated mature plants, as this is preferentially done in the field, such as reported by Weber *et al.* (2012) and reviewed by Araus and Cairns (2014). When evaluating mature plants, studies rarely focus on growth, but rather focus on predicting or measuring yield and yield-related parameters, which are a major objective for crop breeding (Weber *et al.*, 2012; Araus and Cairns, 2014).

Root imaging is inherently difficult in the field (Reynolds *et al.*, 2012; Wasson *et al.*, 2012). ‘Shovelomics’ and similar methods, in which the roots are excavated and analysed, have been proposed as an approach to characterize root system architecture under field conditions, but these methods are time consuming and involve destructive analysis (Trachsel *et al.*, 2011; Bucksch *et al.*, 2014). Few studies have reported root imaging under natural conditions and without destructive harvesting, such as imaging of roots using the Growth and Luminescence Observatory for Roots (GLO-Roots) system (Rellán-Álvarez *et al.*, 2015) or in a more artificial system using transparent growth media (such as gel or glass beads) (Courtois *et al.*, 2013; Topp *et al.*, 2013). Improvements to non-destructive analyses involve not only automated data gathering and data analyses, but also the development of new technologies that allow determination of root parameters as well as the development of models to recover the structures of plants using 3D models (Ward *et al.*, 2015). We believe that these technologies will provide a step change in salinity research, especially when time resolution is incorporated to provide insights into the dynamic responses of plants to salinity. In the future, this will allow the design of new types of experiments that should enable, for example, the monitoring of changes in root architecture in response to salinity treatment.

Whether the destructive or non-destructive approach is chosen will depend on the biological question asked and on access to technologies. Destructive harvests are generally suitable for long-term salt stress experiments (many days/weeks), when the differences in growth parameters, such as biomass, are readily apparent. Non-destructive analyses, such as imaging, allow the monitoring of the same plant over multiple time points, such as before and after stress application, enabling the detection of small and dynamic differences in growth parameters. By following the growth of each plant throughout the experiment, it is possible to separate the effects of salt on the inhibition of the production of new leaves from the acceleration of senescence and death of old leaves.

A number of indices can be derived from biomass measurements (Supporting Methodologies Section 2). For instance, a commonly used index to compare different accessions and even species is the salt tolerance index (ST), calculated as the percentage of biomass production over a defined period under saline compared with non-saline conditions (Munns *et al.*, 2002) [Supporting Methodologies Section 2, eqns (c) and (d)]. The tolerance index (TOL) measures the difference in biomass production between salt-treated and control conditions (Rosielle and Hamblin, 1981) [Supporting Methodologies Section 2, eqn (e)]. The stress tolerance index (STI) takes into account both the overall biomass production of the population under control conditions and the ability to maintain yield (or other growth parameters) under stress conditions, favouring the selection of genotypes that perform well under both control and stressed conditions (Fernandez, 1992) [Supporting Methodologies Section 2, eqn (f)].

A recognizable indication of salinity stress is a reduction in shoot growth, which, in turn, can change the allocation of biomass between roots and shoots. This change can be described using the root mass ratio (RMR). A lower relative RMR indicates that a plant reduces allocation of biomass to the roots upon salt stress to a greater extent than under control conditions [Supporting Methodologies Section 2, eqns (g)–(i)].

Salinity stress also affects cell expansion in young leaves, generally causing a decrease in leaf area (Munns and Tester, 2008). The leaf area ratio (LAR) is the ratio of leaf area to the leaf’s dry mass. Relative LAR (RLAR; the LAR under saline compared with control conditions) provides a measure of the effect of salinity on what is effectively leaf thickness. A reduction in RLAR under salinity stress may be adaptive, given the leaf’s thicker cell walls or perhaps greater volume into which salts could be sequestered [Supporting Methodologies Section 2, eqns (j)–(l)].

### Quantifying the effects of salinity on plant water relations, transpiration and transpiration use efficiency

The effects of salinity stress on a plant’s water relations have been described previously in the classical literature (Munns and Passioura, 1984; Nobel, 1991). Two components of a plant’s water relations are water potential and hydraulic conductivity. Water potential refers to the potential energy of water relative to pure water, and therefore determines the direction of water movement, where water moves from a location with a higher water potential to a location with a lower water potential. Hydraulic conductivity refers to the ease with which water can flow from one location to another and therefore affects the rate of water movement. In the face of high salinity, a plant’s ability to control these two components is essential. Two additional components, in combination with other components, are the outputs of water potential and hydraulic conductivity, namely the maintenance of water levels in tissue (the primary determinant of cellular growth and function) and the maintenance of transpiration [T; along with transpiration use efficiency (TUE), a related component]. Both enable a plant to continue to grow. In this review, we briefly introduce water potential and hydraulic conductivity. We then focus on the maintenance of water levels and transpiration because of their myriad physiological consequences and because their high-throughput measurement is now possible.

To learn about the many methods used to quantify the energy levels of water in plants, the reader may consult an extensive classical literature on this subject, including many papers by Munns and Passioura (e.g. Munns and Passioura, 1984; Passioura and Munns, 2000; Nobel, 1991) and books by Nobel (2005) and Meidner and Sheriff (1976). General undergraduate textbooks such as that by Taiz *et al.* (2015) also treat this topic well.

It has been argued that salt-tolerant plants decrease the hydraulic conductance of their roots, thereby reducing the delivery of (salty) water to the shoot (Vysotskaya *et al.*, 2010) and resulting in reduced water potential in their leaves (Gama *et al.*, 2009). While it can be informative to assess the effects of salinity on hydraulic conductance in the roots using a high-pressure flow meter (following, for instance, the method of Tyree *et al.*, 1995) or on water potential in the leaves using a pressure chamber (following, for instance, the method of Scholander *et al.*, 1965), these are specialized measurements that are probably best made in laboratories skilled in such technologies.

The water fraction (WF) of a tissue can be assessed simply. WF is the water content of the shoot (under controlled conditions) as a fraction of the fresh mass of the shoot. In the context of this review, a plant with a higher relative water fraction (RWF, the WF under stress conditions relative to control conditions) is better able to maintain its water content in the shoot upon salt stress [Supporting Methodologies Section 2, eqns (m)–(o)].

Another related trait important to plant function is the ability to maintain water content in tissues at optimal levels in the face of environmental stress. Plants under stress often lose some water from their tissues, which can have rapid and large effects on cell expansion, cell division, stomatal opening, abscisic acid (ABA) accumulation, etc. (Hsiao and Xu, 2000). Most of these effects become evident with no change in turgor pressure, although water potential can become more negative due to osmotic potential becoming more negative (‘osmotic adjustment’ – the ability to change the osmotic potential by alteration of the concentration of salts and/or neutral solutes, thus reducing changes in pressure potential). Most of these effects can also become evident with very small reductions (<10 %) in tissue water content. Here we are describing relative water content (RWC), which has also been extensively used in the classical literature to determine the water status of a shoot relative to its fully hydrated state. Under saline conditions, plants usually adjust their osmotic potential to maintain turgor pressure and this can exacerbate difficulties with classically used methods to measure RWC (Boyer *et al.*, 2008). As such, quantifying the effects of salinity on RWC is important and physiologically relevant. Methods to do this are included in Supporting Methodologies Section 2, eqn (p).

It has been proposed that the ability of plants to maintain normal rates of transpiration under saline conditions is an important indicator of salt tolerance, particularly because transpiration is related to normal rates of CO_2_ uptake for photosynthesis (Harris *et al.*, 2010). However, assessment of a plant’s transpiration rate using porometers (Meidner and Sheriff, 1976) and infra-red gas analysers (Nobel, 1991) can be difficult due to rapid changes in stomatal conductance that can occur in both space and time (Munns *et al.*, 2006). Thus, measures of T and TUE depend on the leaf, time of day and the particular part of the leaf from which the measurements are taken. In this review, we discuss T and TUE at the whole-plant level. Methods to measure T and TUE are described in Supporting Methodologies Section 2, eqn (q).

TUE is dependent on both the genotype and the environment (Vadez *et al.*, 2014). The genes involved in TUE remain largely unknown (Masle *et al.*, 2005), and the effects of salinity on TUE remain, to our knowledge, largely unstudied. Carbon isotope discrimination has been used for analysis of TUE (Farquhar *et al.*, 1982), and it has been used successfully to improve the water use efficiency of wheat (Farquhar *et al.*, 1982; Condon *et al.*, 2004). We note that the tissue sampled for measurements of carbon isotope discrimination must be carefully chosen to obtain fair and relevant measures of TUE and the effect of salinity on TUE.

### Quantifying the effects of salinity on ion relations

Maintaining ion homeostasis can be particularly challenging for plants under saline conditions, as the accumulation of toxic ions (i.e. Na^+^) can perturb the plant’s ability to control accumulation of other ions. In most species, Na^+^ appears to accumulate to toxic levels before Cl^−^ does; thus, we focus here on Na^+^, because reducing Na^+^ in the shoot, while maintaining K^+^ homeostasis, is a key component of salinity tolerance in many cereals and other crops. However, in some perennials, Cl^−^ accumulates in the shoot and inhibits photosynthesis whereas Na^+^ appears to be preferentially retained in the woody roots and stems (Flowers and Yeo, 1988).

Most research on salinity estimates the amount of elemental Na and K at the whole-tissue level (leaves or roots) using techniques such as atomic absorption spectroscopy (AAS), flame photometry (FP) or inductively coupled plasma mass spectrometry (ICP-MS); at the sub-cellular level, techniques such as X-ray microanalysis or secondary ion mass spectrometry (SIMS) can be employed (for a complete review of methods, see Conn and Gilliham, 2010). It should be clarified that the abbreviation Na, rather than Na^+^, is used deliberately here because these techniques generally quantify the element and not the ion.

It should also be noted that the Na or K concentration is the amount of the element per unit of volume (e.g. mm) whereas content refers to the amount of the element per unit of mass (e.g. μmol g^−1^ dry mass). There is confusion in the plant science literature over use of the term ‘content’, and we need to standardize this as a community to be in line with international commonly accepted usage in all other fields of science. The use of the term ‘content’ in other fields is clear – it is reserved for the amount per unit mass (Tolhurst *et al.*, 2005; Dybkaer, 2007; Fuentes-Arderiu, 2013). Crucially, the Bureau International des Poids et Mésures (BIPM), the international organization of metrology, use the term ‘content’ in this way, such as can be seen at http://goo.gl/pEqQHW. Content can be calculated as the amount per unit mass (amount-of-substance content, with SI units of moles per gram) or as the mass per unit mass (which can be termed the ‘mass fraction’). The confusion in the plant literature arises from the use of content for the total amount of a substance in a particular organ – as opposed to the concentration. However, we suggest that the community use the term amount for this (with units of moles or kg), and express it as amount per organ.

We recommend calculating Na and K concentrations (i.e. in mm), rather than Na and K content (i.e. μmol g^−1^ dry mass) because the latter does not account for differences in the water status of tissues (particularly if one is comparing different accessions or species), and thus differences in concentration in the aqueous phase, which is the primary factor directly affecting transport and biochemical processes, might be missed. Of course, this does not apply in tissue that has visible significant signs of senescence and desiccation.

It is also beneficial to measure the amount of Na and K in the roots of the plant, as this will indicate how much Na^+^ is retained in the roots. Hydroponically grown plants are particularly suited for ion analyses as soil particles do not interfere with the collection of root material and ion analysis. Care must be taken to quantify Na and K in plant roots. Roots need to be rinsed for a short time with a Ca^2+^ solution to remove apoplastic Na^+^ (e.g. as in Davenport and Tester, 2000) to prevent damage to cells. It is also good practice to adjust the osmotic potential of the rinse solution to equal that of the growth solution, to prevent cell damage and remove the need for tissues to osmotically adjust. More details on choosing and processing samples for ion analysis are provided in Supporting Methodologies Section 1.

It has been proposed that the salinity tolerance of a plant is determined not only on the basis of the leaf Na^+^ concentration, but also on the ability of the plant to maintain high cellular K^+^ levels (Shabala and Cuin, 2008). It is therefore common to present the Na/K ratio for both roots and shoots; however, this ratio is affected mainly by changes in the Na^+^ concentration, which are commonly proportionally much greater than changes in the K^+^ concentration.

Ions that accumulate during salt stress can be compartmentalized into different types of cells in a particular organ. For example, X-ray microanalysis (a semi-quantitative method that identifies relative ion locations) of salt-stressed barley leaves showed that the ion composition in vacuoles of mesophyll cells differed from that of vacuoles of epidermal cells (Leigh and Storey, 1993).

The classical method for analysing ion fluxes within plants is the pulse-chase experiment, which involves exposing plants to radioisotopes such as ^22^Na^+^, ^36^Cl^−^ or ^42^K^+^ (it is risky to use ^86^Rb^+^ as tracer for the flux of K^+^ because its behaviour can often differ markedly from that of K^+^, especially when making comparisons with Na^+^ flux). Although technically challenging, pulse-chase techniques are powerful for studying the unidirectional fluxes of Na^+^ and K^+^ into roots and the movements of ions in plants subjected to salinity stress (Davenport *et al.*, 2007). It is important to note that the unidirectional flux of Na^+^ into roots appears to be quite high, requiring measurement over very short times (on the order of 5 min); otherwise, the efflux of the radioactive tracer begins to become significant, reducing the apparent rate of influx (reviewed by Tester and Davenport, 2003). Net fluxes can also be calculated by measuring increases in tissue ion concentration using sequential harvests. In addition, the use of extracellular vibrating ion-sensitive electrodes can be very useful for measuring net fluxes of ions (Shabala *et al.*, 2013), as can changes in bulk concentrations in either external solutions or plant tissues upon a step change in external concentrations. These approaches, however, cannot be used for measuring unidirectional fluxes, and also have some limitations because of issues with selectivity of resins used in electrodes (e.g. of Na^+^-selective ionophores) and also the ability to detect depletion of ions when external ion concentrations are high (such as in saline solutions).

Accumulation of compatible solutes, such as glycine betaine, proline and polyols, in the cytoplasm is required to balance the decrease in water potential occurring in the vacuole due to ion accumulation in that compartment (dos Reis *et al.*, 2012). It is well established that compatible organic solutes increase with salt stress; however, whether a greater increase in compatible solutes correlates with increased salinity tolerance in plants remains to be shown. For barley, at least, it appears that the more salt-tolerant varieties accumulate less compatible solutes than do the more sensitive varieties (Chen *et al.*, 2007). Concentrations of glycine betaine, proline, polyols and sugars can be quantified using techniques such as high-performance liquid chromatography (HPLC) with UV detection (Naidu, 1998; Abraham *et al.*, 2010) or gas-liquid chromatography (GLC) methods (Holligan, 1971). In some cases, as for proline, simpler methods have been established, such as a ninhydrin-based colorimetric assay (Abraham *et al.*, 2010). A simple method to quantify sugars is a colorimetric assay using anthrone (Yemm and Willis, 1954).

### Quantifying the effects of salinity on photosynthesis

Upon salinity stress, a substantial decrease in a plant’s stomatal aperture can be observed, but the rates of photosynthesis per unit leaf area sometimes remain unchanged (Munns and Tester, 2008). Previous work using two contrasting durum wheat genotypes showed that salinity stress caused a large decrease in stomatal conductance (*g*_s_) of both genotypes (James *et al.*, 2002). Interestingly, the efficiency of photosystem II (PSII) in the tolerant wheat accession was unaffected, while there was a decline in the quantum yield of PSII photochemistry, coinciding with leaf ageing, higher Na^+^ and Cl^–^ concentrations in the leaf, and chlorophyll degradation, in the sensitive genotype (James *et al.*, 2002). Following stomatal closure, the internal reduction of CO_2_ decreases the activity of several enzymes including RuBisCo (Chaves *et al.*, 2009), thus limiting carboxylation and reducing the net photosynthetic rate. The intercellular CO_2_ concentration (Ci) is another parameter that has been used to estimate the effects of salinity on photosynthesis (Seemann and Critchley, 1985; Redondo-Gomez *et al.*, 2007; Stepien and Johnson, 2009). Under salinity, the CO_2_ assimilation rate (as a function of Ci) was shown to be better maintained by a salt-tolerant species, *Eutrema salsugineum*, compared with a sensitive-species, *Arabidopsis* (Stepien and Johnson, 2009). However, it is difficult to differentiate cause–effect relationships between photosynthesis (source) and growth reduction (sink); also, the effects of salinity on photosynthesis can be caused by alterations in the photosynthetic metabolism, or else by secondary effects caused by oxidative stress (Chaves *et al.*, 2009).

To understand the impact of salinity on photosynthetic responses, many studies quantify the amount of chlorophyll in the leaf (expressed, for instance, as μg Chl g^−1^ tissue or μg Chl cm^−2^ tissue) (Arnon, 1949; Hiscox and Israelstam, 1979). However, under salinity stress, leaf expansion, associated with changes in leaf anatomy (smaller and thicker leaves), is reduced, resulting in higher chloroplast density per unit leaf area, which can lead to a reduction in photosynthesis as measured on a unit chlorophyll basis (Munns and Tester, 2008). Non-invasive methods that capture photosynthetic responses include measurements by infrared gas analysers (IRGAs) and pulse amplitude-modulated (PAM) chlorophyll fluorometers. In addition, the use of soil and plant analyser development (SPAD) meters to determine the chlorophyll content can also provide an estimate of leaf damage under stress. The chlorophyll content can be estimated using the SPAD index, which is the ratio between leaf thickness (as determined by the transmission of light in the IR range) and leaf greenness (as determined by the transmission of light in the red light range). The SPAD index has been shown to decrease under salinity compared to control conditions. The extent of this decrease has been shown to vary between barley accessions, suggesting a genetic control of this effect of salinity on the SPAD index (Adem *et al.*, 2014). It should be noted that the interpretation of SPAD meter measurements is not straightforward because salinity stress can increase leaf thickness (Longstreth and Nobel, 1979) and thus influence SPAD meter readings in a way that is independent of effects of salinity on chlorophyll content (Li *et al.*, 2009). Therefore, careful calibration of the system, as well as the use of species-specific calibration equations, are necessary to determine chlorophyll content (for further details the reader is referred to Richardson *et al.*, 2002). Also, SPAD meter measurements should be carried out at the same time of day to avoid variation due to diurnal changes, and they should be performed on the same leaf and the same location on the leaves of every plant to reduce effects of spatial variation. Several studies have shown the existence of genotypic differences in photosynthetic responses due to salinity (James *et al.*, 2002, 2008; El-Hendawy *et al.*, 2005). To study the effects of salinity on the regulation of photosynthesis, consistency in the measurements is essential. Such measurements are dependent of the time of day, which leaf is measured and the position on the leaf where the measurements are taken. Extensive replication is required to obtain a representative measurement for a particular genotype. This, in turn, reduces capacity for comparative measurements.

Thermal imaging using IR thermography has also been used as an indication of stomatal regulation in response to abiotic stress (Jones, 1999). In barley plants subjected to salinity stress, there is a strong relationship between direct measurements of stomatal conductance and leaf temperature and these differences are dependent on genotype (Sirault *et al.*, 2009). IR thermography measurements may be most profitably used to assess the early response to salinity stress (osmotic phase), before other plant processes confound the measurements, such as the build-up of salt in the leaves, causing changes in leaf morphology, and before age-associated decreases in stomatal conductance occur (James and Sirault, 2012). IR thermography should therefore be completed on young seedlings (leaf 2–3 stage for cereals), shortly after the final desired concentration of salinity is attained (at 3–5 d) (James and Sirault, 2012).

### Quantifying the effects of salinity on plant senescence

Once the plant has accumulated Na^+^ in the shoot and suffers from the toxic effects of Na^+^, the most visible symptom is a yellowing, then browning, of leaves, due to leaf senescence and death. This effect is most visible in older leaves that have had a longer time to accumulate Na^+^ and suffer from the effects of that accumulation. However, it is notable that the leaves of some plants are better able than others to maintain greenness and photosynthetic function for longer in the presence of high levels of Na^+^ in tissues. The classical way to determine leaf senescence is by using a visual scoring method, which can be used to compare different plant genotypes affected by salinity. Such scoring methods can also be used in combination with growth analyses. An example of the scoring of growth and leaf damage in rice seedlings is presented in Table 1.

**Table 1:** Standard evaluation system of visible salt damage in rice at the seedling stage (Gregorio *et al*., 1997)

Score	Observation
1	Normal growth, no leaf symptoms
3	Nearly normal growth with some leaves and tips whitish and rolled
5	Growth severely retarded with most leaves rolled and only a few elongated
7	Complete growth arrest with most of the leaves dried and some plants dead
9	Almost all plants dead or dying

Senescence can also be estimated in an automated set-up using, for instance, high-throughput fluorescence imaging. Plants are imaged after they have been exposed to salinity for an extended period when clear symptoms of Na^+^ toxicity are visible. This imaging analysis allows calculation of the area affected by salt-induced senescence (SIS) (Rajendran *et al.*, 2009; Berger *et al.*, 2012) [Supporting Methodologies Section 2, eqn (r)].

Tissue tolerance, at the shoot level, refers to the ability of plants to maintain tissue function in the face of high accumulation of Na in older leaves, where leaf senescence is observed first (rather than in younger leaves). SIS, however, is estimated at the whole shoot level; thus, SIS is not an ideal indicator for tissue tolerance. Consequently, to estimate tissue tolerance, measurements of senescence need to be made specifically in older leaves. One way to do this is to use image analysis models to separate individual leaves and to therefore enable measurements of parameters of each single leaf, rather than just those of the whole shoot (Ward *et al.*, 2015). In effect, a full life history of each leaf could be developed, and the effects of salinity and genetic composition on that life history could be quantified. Such a procedure would allow the progression of senescence to be observed in a specific leaf (e.g. leaf 3 in cereals) and thus provide a more accurate estimation of tissue tolerance.

### Quantifying the effects of salinity on yield-related parameters

The ultimate goal of salinity tolerance research is to increase salinity tolerance in crops for them to maintain yield under adverse conditions. Given that research conducted using pots/greenhouse conditions does not provide a reliable estimation of yield responses, fieldwork needs to be undertaken to quantify yield and yield-related parameters (yield components). Supporting Methodologies Section 1 provides further details about the choice of an appropriate experimental system. Soil salinity in the field is not only determined by the concentration of Na^+^ and Cl^−^, but also other ions such as Mg^2+^, Ca^2+^ and ${\text{HCO}}_{3}^{-}$. In the field, soil salinity is often reported as electrical conductivity (EC_a_), which can be determined using instruments such as electromagnetic (EM38) soil mapping devices (Corwin and Lesch, 2013). Field trials require control (low salinity) and saline plots, with a level of stress depending on the species and the available irrigation. The use of check plots with a known genotype adapted to the region is a prerequisite, as is some degree of replication and accounting for spatial variation. Moreover, field trials should be replicated over at least two years to account for heterogeneity in the field and other environmental factors. Heterogeneity in field salinity is a significant issue in field trials in dryland environments; using irrigated fields can reduce spatial heterogeneity significantly, and irrigation with fresh and brackish water can be effective, at least on sandy soils.

Although plants’ sensitivity to salinity is higher during early seedling stage and reproductive stage, crops need to maintain functions at all stages of their life cycle to increase their ability to maintain yield under high salinity. For instance, yield can also be reduced during the vegetative stage by affecting parameters such as tiller number per plant in cereals such as rice (Zeng and Shannon, 2000) or wheat (Maas *et al.*, 1994). The harvest index (HI) has been shown to be affected by salinity (Gholizadeh *et al.*, 2014). A plant capable of maintaining HI under stress conditions will often have a higher yield [Supporting Methodologies Section 2, eqn (s)]. The reason for the maintenance of HI under salinity is not fully understood. Plausible reasons for changes in HI may include a lower shoot biomass reduction, maintenance of tiller number (Zeng and Shannon, 2000) or earlier flowering (Saade *et al.*, 2016). Besides HI, other parameters, such as yield and yield components including seed/fruit mass, spikelets per spike (for cereals), spike length, fertility rates in the spikes and 1000-grain mass, have also been shown to be affected by salinity stress (Gholizadeh *et al.*, 2014; Mishra *et al.*, 2014). Measurements of yield for crops whose harvestable parts are below ground (such as tubers or modified roots) provide their own challenges, but experts in these crops have well-developed methodologies to address this. The principle that such work should be field-based remains.

### Interpreting the physiological results

After all data collection and analyses are completed, the key question then is how the data should be interpreted to address the question, ‘What are the plants telling us?’ In this review, a couple of methods of data presentation and interpretation that lead to improved data analysis are described.

**Fig. 2.**
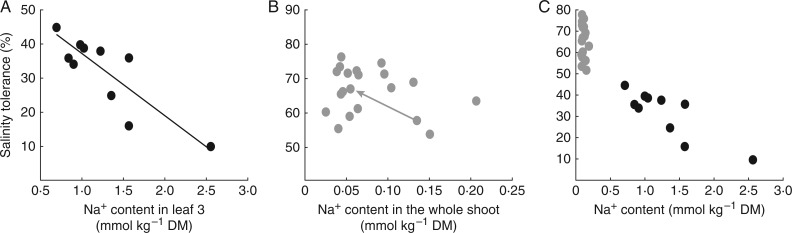
Correlating the salt tolerance index (ST) with Na^+^ content. (A) A strong correlation is observed when plotting ST in relation to Na^+^ content in a number of genotypes of tetraploid wheat, indicating that the more tolerant genotypes accumulate less shoot Na^+^ (modified from Munns and James, 2003). (B) No correlation exists between ST and shoot Na^+^ content in 20 moderately stressed bread wheat varieties (modified from Genc *et al.*, 2007). Although there is no obvious correlation, a decrease in Na^+^ content in shoots may still cause an increase in salinity tolerance, as indicated by the arrow. Note the different *y*-axes, because plants are different species of *Triticum* and were treated with different NaCl concentrations. Note also that values on the *x*-axes were obtained using different, although related, tissues. Genc *et al.* (2007) report a similar lack of correlation when using Na content of just the blade of leaf 3. (C) Data from A and B are plotted on the same axis. Figures used with permission of the publishers.

In the first example (Fig. 2), the salt tolerance index (ST) of different genotypes was plotted in relation to the Na^+^ content in the third leaf or whole shoot (Munns and James, 2003; Genc *et al.*, 2007). A strong correlation between the Na^+^ content of leaves and ST is observed in the genotypes analysed in Fig. 2A, indicating that the Na^+^ content in the third leaf may be associated with salinity tolerance in these genotypes. On the other hand, no correlation is found between ST and the content of Na^+^ in shoots in the genotypes analysed in Fig. 2B. It is noteworthy that the lack of correlation in Fig. 2B does not necessarily mean that a particular trait (in this case the content of Na^+^ in shoots) does not play a part in salinity tolerance; this example illustrates that in some genotypes, the Na^+^ content in the shoot can contribute to salinity tolerance (Fig. 2A), while in others salinity tolerance appears to be due more to other factors (Fig. 2B). That Na^+^ exclusion can be important in some genotypes is shown by direct manipulations of Na^+^ accumulation in shoots, such as by simple genetic variation (Munns *et al.*, 2012), or by addition of Ca^2+^, which reduces Na^+^ accumulation in shoots (Lahaye and Epstein, 1969). Although there is no obvious correlation, a decrease in Na^+^ content in shoots may still cause an increase in salinity tolerance, as indicated by the arrow in Fig. 2B.

As shown in Fig. 2, any trait that is hypothesized to contribute to salinity tolerance (e.g. TUE, RMR or HI) may be plotted on the *x*-axis in relation to ST – where salinity tolerance is best measured by the ability to maintain yield in saline conditions relative to control conditions. A correlation between the trait of interest and ST in the analysed genotypes will be an indicator that the trait contributes to salinity tolerance in the plants being tested.

**Fig. 3.**
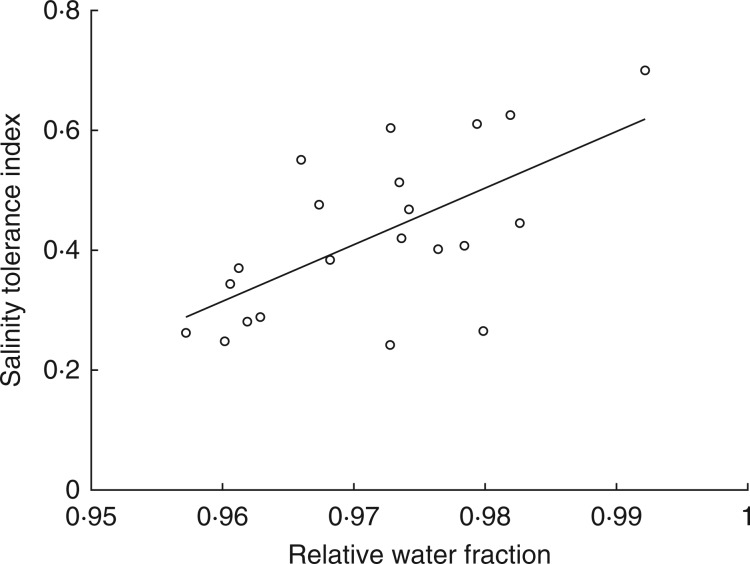
Correlation between salt tolerance index (ST) and the relative water fraction (RWF) in *Arabidopsis thaliana* ecotypes. Plants were grown in hydroponics for 4 weeks according to Conn *et al.* (2013), and subjected to 7 d of salt stress after increasing salinity to 125 mm NaCl over three increments separated by 12 h each. CaCl_2_ was added to the medium to maintain constant Ca^2+^ activity. Unpublished data of Dr David E. Jarvis.

Another example is the positive correlation between ST and RWF as shown in Fig. 3. In this example, different *Arabidopsis thaliana* genotypes were exposed to 125 mm NaCl for 7 d (D. E. Jarvis, KAUST, unpubl. res.). Figure 3 shows the correlation between ST and RWF, indicating that this trait is associated, at least in part, with salinity tolerance. A possible explanation could be that plants that are better able to maintain their water status are more salt tolerant. Of course, these data could also be interpreted from the opposite perspective, i.e. that plants that are more salt tolerant are better able to maintain their water status. Cause and effect can often be difficult to disentangle.

**Fig. 4.**
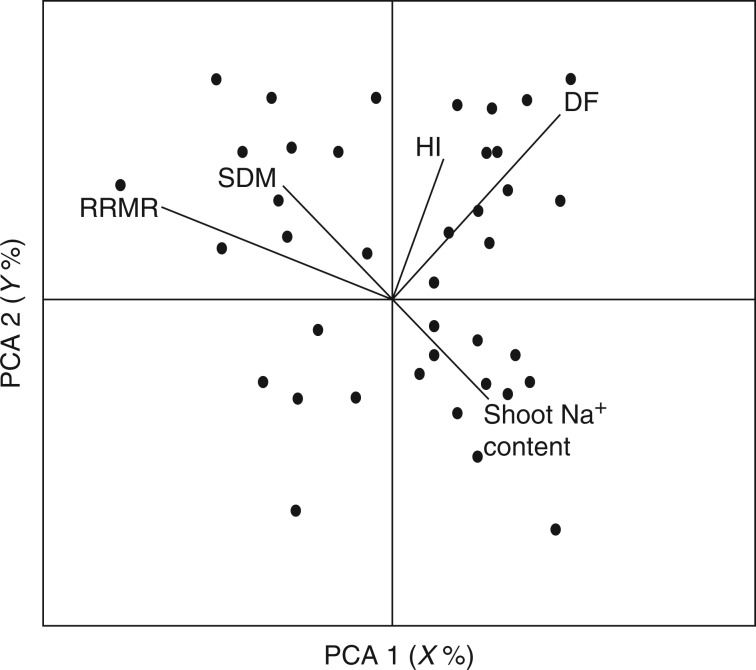
Example of the use of principal component analysis (PCA) to assess the importance of traits contributing to salinity tolerance. The example traits used here are relative root mass ratio (RRMR), shoot dry mass (SDM), harvest index (HI), days to flowering (DF) and shoot Na^+^ content.

Simple correlation analyses (e.g. using pairwise regression) are often preferred, as their calculation is straightforward. However, complex and important traits might be difficult to dissect and a simple pairwise analysis may not yield satisfying results. In this case, data can also be analysed and presented using principal component analysis (PCA) or non-linear PCA. The use of PCA can provide an indication of the most important traits contributing to salinity tolerance in the materials and conditions under study. In the hypothetical example provided in Fig. 4, the distribution of the genotypes shows that PCA1 and PCA2 account for (*X*+*Y*) % of the total variability in the set of variables (traits) analysed in each genotype. In this example, PCA1 accounts for *X* % of the variability and it is strongly negatively correlated with the relative root mass ratio (RRMR) and, to a lesser extent, with the shoot dry mass (SDM). This is in contrast to days to flowering (DF), which is positively correlated with PCA1. DF is therefore a trait that is positively associated with salinity tolerance in this example. The figure indicates that HI, DF and shoot Na^+^ content are the best discriminating parameters for PCA2, explaining *Y* % of the variability. Moreover, traits such as DF and SDM, as well as DF and shoot Na^+^ content, are independent variables, whereas HI and DF are strongly correlated with each other. Under these hypothetical conditions, the shoot Na^+^ content and SDM are negatively correlated, suggesting that plants with lower shoot Na^+^ content have more shoot biomass under saline conditions.

## CONCLUSIONS

A proposed experimental route from design to data analysis should include classical screening of germplasm for breeding purposes, characterization of genes, and discovery of new quantitative trait loci (QTL) and, ultimately, genes using forward genetics.

Yeo *et al.* (1990) have suggested to study salinity tolerance not based on overall performance, but rather to look at traits that contribute to salinity tolerance (such as shoot Na^+^ content or plant vigour). It has been long considered that it is highly unlikely that one gene alone determines plant salinity tolerance, so a useful strategy to obtain salt-tolerant varieties is to pyramid several genes contributing to salinity tolerance (Yeo and Flowers, 1986; Yeo *et al.*, 1990). The effects of salinity stress on plants are complex and results can be difficult to interpret if experiments are not designed carefully and if appropriate measurements are not made. To facilitate the interpretation of results from tests investigating effects of salinity on plants, we propose analyses of salinity responses not at the level of the whole plant (e.g. simply total plant biomass), but rather at the component (or trait) levels that are hypothesized to contribute to salinity tolerance. In the future, the relevance of such traits on maintenance of yield (and quality) under saline conditions can be tested in the field. The assessment of seedling survival or shoot Na^+^ content may not be meaningful as a predictor of salinity tolerance without other information, such as the effect of salinity on various growth parameters. In this review, we have aimed to describe methods used to measure some of the traits that may contribute to salinity tolerance. To allow useful measurements to be performed, we recommend systems and time scales that are appropriate for addressing particular biological questions.

New technologies and methods will improve quantitative comparisons within the same species by including different genotypes, and also of different species, such as *Eutrema salsugineum* with *Arabidopsis thaliana*, domesticated tomato with wild relatives such as *Solanum cheesmaniae*, and domesticated rice with wild relatives. Furthermore, if experimental conditions are accurately documented, comparisons of results obtained from different laboratories should be possible. Constant advances are being made to identify traits that are associated with salinity tolerance, such as measurements of HI and WUE, allowing us to get a better understanding of the complex network of traits that contribute to salinity tolerance. Undoubtedly, the rapid development of bioinformatic tools and high-throughput ‘omics’ platforms will boost the acquisition of physiological data, with consequent benefits to research on salinity tolerance in plants.

## SUPPLEMENTARY DATA

Supplementary data are available online at www.aob.oxfordjournals.org and consist of the following. Figure S1: sensitivity of rice to salt varies over the course of the life cycle [image from Singh *et al.* (2008), with permission of the authors and the publisher]. Table S1: range of salt concentrations advised for use with five species in hydroponic, soil-filled pots and field experiments. Movie S1: growth of wheat leaves decreases during salinity stress. Wheat plants were grown over a 13-d period. On day 9, the plant on the right side was treated with NaCl; the control plant is on the left side. Within 2 d, a clear inhibition of growth is visible in the wheat plant exposed to NaCl.

Supplementary Data
