# Supplementary material for: Evaluating physiological responses of plants to salinity stress
Source: Ann Bot. 2016 Sep 5;119(1):1–11. doi: 10.1093/aob/mcw191 (PMC5218372; doi:10.1093/aob/mcw191)
Supplement: Supplementary Data [file supp_119_1_1__index.html]

Evaluating physiological responses of plants to salinity stress — Supplementary Data 

# Evaluating physiological responses of plants to salinity stress

## Supplementary Data

files

- Supplementary Data - zip file
