# Supplementary material for: Evaluating physiological responses of plants to salinity stress
Source: Ann Bot. 2016 Sep 5;119(1):1–11. doi: 10.1093/aob/mcw191 (PMC5218372; doi:10.1093/aob/mcw191)
Supplement: Supplementary Data [file supp_mcw191_suppl_data.zip › aob-16508-s02.docx]

**Supporting Methodologies**

***Section 1: Key experimental aspects to consider***

A number of aspects should be considered when designing and implementing experiments with plants, as discussed by Poorter, H. *et al.* (2012). For studies that analyse salinity tolerance, the following aspects are particularly important:

1. Ensuring good seed quality and consistency;
2. Choosing appropriate controls;
3. Defining relevant levels of salt stress for the species of interest (testing for maintenance of growth or yield, rather than survival);
4. Determining a suitable developmental stage for imposing salt stress, and also determining appropriate time point(s) for sample analysis;
5. Choosing the appropriate system (e.g. agar plates, hydroponics, pots or field) for the salt stress assay; and
6. Choosing relevant samples for ion analysis.

**A. Seeds**. One important aspect for a successful experiment, often not mentioned explicitly, is that the origin and age of the seeds must be consistent to minimize variation in plant performance due to factors other than salinity. It is good laboratory practice to use seeds derived from plants grown at the same time and under the same conditions, to use recently harvested seed stocks, and to ensure that seeds have been stored under appropriate conditions. It is particularly important to store seeds at low humidity and, if possible, at low temperature (e.g. 4 °C).

Variability in plant performance is reduced if seeds of similar size are used for the experiment. Seed size can impact growth of the plant, especially over the relatively short time period often used for salinity tolerance experiments (Black, 1956). Seed sorting can be done by eye into, for instance, three size groups; then, only the middle-sized set of seeds is used for the experiment. Consideration needs to be given to whether seeds should be cold treated before germination (e.g. by suspending seeds in water and incubating them at 4 °C for 3 d), as such cold treatment makes germination more consistent and therefore beneficial to the experiment. Because cold treatment could affect salinity tolerance, all seeds in the experiment should be cold treated the same way (Taeb *et al.*, 1992; Sharma & Kumar, 1999).

**B. Controls**. When analyzing transgenic lines, not only wild-type plants should be included (for example, the “mother” line) but, more importantly, null segregants from the heterozygous primary transformants should also be included. Null segregants are plants that do not carry the transgene, but have undergone the transformation and selection process along with the transgenic lines. They are generated upon segregation of the transgene in generations grown after the transgenic event. Using plants transformed with “empty vector” constructs, or generating transformants with the gene of interest mutated to an inactive form, also provides good controls. It is prudent to use several controls. We should also mention that it is necessary to include multiple transgenic lines generated from independent transformation events to ensure physiological effects are attributable to the transgene, and not due to other effects, which may arise from the location of the insert in the genome or other mutations or epigenetic changes occurring during the transformation process.

**C. Relevant stress levels**. We should note that several salts can evoke salt stress in plants. In this viewpoint, we focus on NaCl stress, and in particular on Na^+^, because in most species this ion appears to accumulate to toxic concentrations before Cl^-^ and other ions. When designing a salt stress experiment, appropriate salt concentrations should be determined according to the plant species being investigated and the particular experimentation system. Different plant species are known to tolerate different levels of salt stress (Munns & Tester, 2008). As an illustrative example, a rice plant grown hydroponically typically shows signs of significant Na^+^ toxicity when exposed to only 50 mM NaCl; a barley plant grown hydroponically, by contrast, can usually tolerate 100 mM NaCl with few or no symptoms of Na^+^ toxicity. When selecting concentrations of salt stress, it is crucial to consider the purpose of the experiment and whether it is survival or tolerance that is being compared. When measuring tolerance traits, the selection of a moderate stress that will allow the plant to complete its life cycle is crucial to enabling the comparison of biomass and/or yield. Examples of different stress levels that are best used for different species are presented in Supplementary Table 1.

It is also important to stress plants moderately to levels that are likely to be relevant to future applications in the field. A farmer is unlikely to grow a crop if its growth is reduced to one-third of its potential due to salinity; studies in which plants are stressed to this extent are less likely to provide information of relevance to future applications. This is important, as mechanisms that have significant impact on tolerance at moderate salt stresses are likely to differ from those that have significant impact on tolerance at higher stresses.

**D. Timing**. Plant age and developmental stage are additional important factors to consider, as a plant’s sensitivity to salt stress is often dependent on the growth stage. For instance, rice is more sensitive to salt stress at both the seedling and reproductive stages than during the main period of vegetative growth (Figure 1). Consequently, in cereals such as wheat, barley or rice, salinity stress can be usefully imposed at the third leaf emergence stage (Munns & James, 2003). For hydroponically or soil-grown Arabidopsis, salinity stress and sampling should be completed during vegetative growth to avoid interfering with processes likely to occur in the reproductive phase. For instance, when plants are grown under short day conditions, the salt stress can be imposed at the tenth leaf stage (also referred to as stage 1.10 according to (Boyes *et al.*, 2001)) and samples can be harvested after a stress period of 7 d.

In practice, complications arise when the experiment aims to compare different genotypes with different growth rates, and particular care should be taken so that the salt stress is imposed at a similar developmental growth stage, and not according to plant age. Even more challenging can be the comparison of genotypes with considerably different growth rates. On one hand, a faster growing plant is likely to have a higher transpiration rate and consequently greater transfer of salt to the shoot through the transpiration stream. It will therefore have to cope with more salt compared to a slow-growing plant with less transpiration. On the other hand, this will be balanced by having a larger volume of tissues into which salt can be sequestered. For instance, the growth rate of *Arabidopsis thaliana* is greater than that of *Eutrema salsugineum* (formerly *Thellungiella halophila*); however, the growth rates of different lines can be brought closer together by adjusting the light regime (Volkov *et al.*, 2004). In some cases, it may be beneficial to design experiments with a single time point for measurements. Different growth rates can then be compensated by planting at different times.

**E. An appropriate system**. Researchers should select the system that is most appropriate for the question being addressed. If, for example, traits in young plants are being studied, then the use of hydroponics or pots may be most suitable; if yield data are of greatest interest, then field experiments should be conducted.

A number of systems for studying salinity tolerance have been used over the years, including a) agar plates, b) hydroponics, c) soil-filled pots, and d) field experiments. Agar plates and hydroponics are often used for model plants such as Arabidopsis, whereas hydroponics, pots and field systems are more frequently chosen to assess cereal and other crop plants. All systems have advantages and limitations for salinity studies, as outlined below:

1. Agar plates

The agar plate system entails growing plants on solidified nutrient media in sealed Petri dishes. This system is particularly popular, as it is easily set up, takes little space and NaCl concentrations can be accurately delineated through addition of NaCl directly to the nutrient media. As agar is translucent, this system is suitable for plant imaging. It is therefore often used to study the effects of root growth and root architecture in response to salinity (e.g. Armengaud *et al.*, 2009; Duan *et al.*, 2013; Julkowska *et al.*, 2014). The agar plate system is also amenable to high-throughput instrumentation and is thus often used to study the effects of salinity on germination. However, this system constitutes a closed environment in which evaporation is reduced and transpiration is restricted. This is important, because the transpiration stream mediates the translocation of ions from the root to the shoot. Hence, plants growing under these conditions are likely to be more sensitive to the ion-independent effect (or “osmotic” stress) in the shoots as a result of the reduced transport of Na^+^ to the shoot (Tester & Davenport, 2003). In addition, leaves often touch the agar, which provides a direct path for Na^+^ entry into the leaves, “short-circuiting” the normal root-based controls for Na^+^ accumulation in the shoot. For these reasons, Na^+^ accumulation in shoots or biomass traits should be assessed using other systems, such as hydroponics or soil-based systems, where plants can transpire and the shoot does not come in direct contact with the salt.

1. Hydroponics

Systems for hydroponically grown plants have been described in detail for Arabidopsis (Conn *et al.*, 2013) and for cereals such as barley (Shavrukov *et al.*, 2012), wheat (Genc *et al.*, 2010), and rice (Gregorio *et al.*, 1997). As in the agar plate system, the concentration of salt can be well defined in the hydroponics system through addition of NaCl directly to the nutrient media. The major advantage of this system over the use of agar plates is that plants are grown under transpiring conditions; hence, salt is translocated in the transpiration stream to the shoot, which allows assessment of salinity tolerance traits such as biomass and Na^+^ content in the shoots. Furthermore, the absence of interfering soil particles enables certain root traits to be analysed.

A number of optimal nutrient solutions have been developed to cater for differing species-specific nutrient requirements. It is important to note that addition of NaCl to a nutrient solution changes the activity of several nutrients, such as calcium (Ca^2+^), magnesium (Mg^2+^) and phosphate (PO_4_^3-^), thus altering their availability to the plant. Of particular importance here is maintenance of the Ca^2+^ activity, because hydroponic solutions have a low nutrient buffering capacity, which can cause plants to develop symptoms of nutrient imbalance (Genc *et al.*, 2010). The well-known interactions between Na^+^ and Ca^2+^ make the impact of NaCl on Ca^2+^ activity a particularly important consideration (Epstein, 1961; Davenport & Tester, 2000a). Moreover, when Na^+^ is present at high concentrations, it can displace membrane-associated Ca^2+^, thus causing Ca^2+^ deficiency (Cramer *et al.*, 1985). This Ca^2+^ deficiency can be alleviated by the addition of supplemental Ca^2+^ to the solution (Lahaye & Epstein, 1969; Cramer *et al.*, 1987). Chemical speciation programs, such as Geochem-EZ (Schaff *et al.*, 2010) or Visual MINTEQ (http://www2.lwr.kth.se/English/OurSoftware/ Vminteq/), can be used to gain an understanding of the physical and chemical interactions between the nutrient solution components and to calculate the optimal species-adapted nutrient solution.

Another important factor to take into account when using hydroponics is the use of incremental salt applications to ensure that plants are subjected to salt stress and not to salt shock (Shavrukov, 2013). In Arabidopsis, increments of 25 mM NaCl have been used 12 h apart until a final NaCl concentration of 100 mM is reached (e.g. Jha *et al.*, 2010); in wheat in supported hydroponics, daily increments of 25 mM have been used until a final concentration of 100 mM is reached (e.g. Genc *et al.*, 2007). While NaCl is the predominant salt used to assess plant salinity tolerance in a hydroponics system, we should also mention that in soils and in irrigation water other cations can also be present, such as Ca^2+^ and Mg^2+^, as well as other anions such as sulfate (SO_4_^-2^) and bicarbonate (HCO_3_^-^). The effects of these other ions could also be further investigated in salinity tolerance.

Keeping roots well aerated is particularly important for salinity experiments, because oxygen supply to the root has a very significant effect on salinity tolerance, probably because it has a large effect on shoot ion accumulation (Malik *et al.*, 2009). Nevertheless, care must be taken when regulating the air flow because the movement of the nutrient solution may cause physical damage to the roots in some species (Wetson & Flowers, 2010). To avoid development of an oxygen-depleted environment around the plant’s roots, some hydroponics systems use an aquarium pump system (bubblers connected to aquarium pumps) to ensure a continuous air (oxygen) supply (Shavrukov *et al.*, 2012).

Another means to deliver oxygen to the roots involves a flood and drain system, whereby the nutrient solution is pumped and then drained off the roots at regular intervals, for example, every 20 minutes. These aerated systems should be used for most plants. Hydroponics systems incorporating the flood and drain system require an inert material, such as polycarbonate plastic beads, to support the root structure and maintain a moist environment around the roots during the drain cycle (Shavrukov *et al.*, 2012). There are significant advantages to using supported hydroponics over standard hydroponics. One problem with normal hydroponics is that, as aerating bubbles break the surface of the nutrient solution, a fine aerosol delivers, over time, a significant amount of salt to the shoot. This does not occur with supported hydroponics. Growing roots in hydroponics also alters root morphology; most notably, it reduces the frequency of root hairs (Graves, 1992). In contrast, supported hydroponics systems provide a solid substrate into which the roots grow, which promotes root development more akin to that for plants growing in soil. A good protocol for screening for salt tolerance using supported hydroponics is detailed in PrometheusWiki (<http://prometheuswiki.publish.csiro.au/tiki-custom_home.php>).

It is also worth noting that rice is a species that is tolerant to submergence. It has aerenchyma to facilitate transport of oxygen to the roots. Consequently, rice does not require an aerated hydroponics system and, given that most rice (and most salt-affected rice) is grown under flooded conditions, it probably should not be grown under aerated conditions. However, the solution in which most hydroponics rice experiments are conducted is aerated.

1. Soil-filled pots

As an alternative to hydroponics, experiments can be conducted using pots filled with soil placed in growth chambers or greenhouses. If pots are filled with other inert solid materials, such as glass pearls or plastic beads, the system is often referred to as supported hydroponics (see above). The use of soil-filled pots increases the complexity of the system due to, for instance, the soil’s matric potential (which describes the interaction between the soil particles and the solution), ion exchanges, and the soil’s microbial environment. The soil forms cation exchange complexes affecting the relative activities of cations and anions in the soil, hence influencing the soil’s water potential and influencing water uptake by the plant. However, soil-based systems are among the most likely to approximate many aspects of field conditions. For example, plants growing in soil will have to cope with high salinity levels plus the effects of the soil’s matric potential, whereas water uptake in hydroponics systems is affected mostly by the ion content and osmotic potential of the nutrient solution (Tavakkoli *et al.*, 2010). The results of previous salinity studies on barley plants grown in hydroponics and soil-based systems have differed in growth, water content and ionic compositions (Tavakkoli *et al.*, 2010; Tavakkoli *et al.*, 2012). It has been recommended that pots should be sufficiently large to ensure a plant-biomass-to-pot volume ratio not larger than 2 g L^-1^ (Poorter, Hendrik *et al.*, 2012). However, this is not universally applicable, as many crops do not enjoy such a generous volume of soil when grown in the field. If Australian wheat varieties are grown in pots with this ratio, they have increased tillering and look and behave differently than do the same genotypes grown in a commercial field. The normal state for many crops is one where root growth is, in fact, restricted, as if in a relatively small pot.

The application of defined salt stress to plants grown in soil is not trivial. There are two main methods for the application of salt stress: soaking the pots in saline water (which has a defined NaCl concentration) or adding a calculated amount of saline water to reach a defined final [NaCl] in the soil. In the first method, pots are soaked in a saline solution of a defined NaCl concentration for a defined time (e.g. 300 mM NaCl for 30 min). However, the final concentration of NaCl in the soil will be unknown, because the residual water in the soil will dilute the added NaCl solution. Also, this method is not replicable if different amounts of soil are used (e.g. due to different pot sizes or varied filling levels), and if the residual water in the pots is different (e.g. due to different evaporation or transpiration rates or different watering regimes). To obtain the defined final [NaCl] in the soil, salt should be added based on the water content in the soil. For this, the water holding capacity of the soil should be determined and the water content at which the plants grow should be established (e.g. at 70% of the water holding capacity). Then, based on the mass of the soil, a defined volume of NaCl stock solution can be added to obtain the desired final [NaCl] in the soil. For instance, if the calculated water content of a given soil in a given pot is 50 g and if the final content should be 100 g of water, then 50 mL of a 300 mM NaCl stock solution can be mixed into the soil to obtain a final [NaCl] of 150 mM. It is beneficial to use draining pots for the soaking method, while non-draining pots are preferable for adding a defined volume of salt solution. For many experiments, such as evaluating the accumulation of Na^+^ in the shoots of an Arabidopsis population, the soaking-method is adequate, as long as all other parameters (such as pot size, filling volume and watering regime) are kept constant.

1. Field experiments

Ultimately, the goal of many research projects is to develop a crop that will produce a yield despite a deleterious environment. This environment is usually a field. (The notable exception to this is hydroponically grown horticultural crops, such as tomatoes.) Moreover, field studies are essential to validate the role and test the utility of specific QTL/gene(s), because a plant that exhibits improved salinity tolerance in the growth room or greenhouse may not necessarily produce improved yield under stress in the field. To assess the practical salinity tolerance of a crop, field experiments are inevitable.

Carrying out a salinity tolerance experiment in the field is not a trivial task. In the field, soil salinity is often spatially complex, not only across the area planted, but also through the soil profile and over time. Additionally, crop yield is also affected by the dynamic properties of soil and other aspects of the environment, which can lead to large variations between field trials in different years and even within one year (Rengasamy, 2006; Corwin & Lesch, 2013). Major components of variation, besides soil salinity, include the heterogeneity of the soil (even just a few metres apart) and environmental factors, which are often difficult to control, such as weather, other abiotic stresses and numerous biotic factors (pathogenic fungi, birds, mice, etc.). For researchers interested in testing field sites for salinity tolerance, the International Biosaline Center for Agriculture (ICBA) (<http://www.biosaline.org/Default.aspx>) presents one of the best examples for yield-salinity trials. ICBA is located in Dubai where it rarely rains, and deep, sandy, homogenous soils are comparatively easy to manage by irrigation, allowing a more even distribution of salinity across the field and down the soil profile. Different salinity levels can be applied through the irrigation water, and because the soil is sandy, use of an appropriate leaching volume can prevent the build up of salts in the root zone (Rhoades *et al.*, 1999).

**F. Analyzing ion content**. The ion composition of plant tissues, e.g. Na, Cl and K content, is a very important trait that is often measured in salinity studies. Some researchers perform ‘whole shoot analyses’, which will miss many physiological processes inside the shoot, since the ions will be recorded as an average for the whole shoot, and within-shoot differences will be missed (e.g. older leaves can accumulate more Na^+^, which will protect younger leaves). Thus, it is important to specify how these analyses are performed to understand ion distribution in terms of compartmentation into organs. In cereal crops, such as barley or rice, the stress is often imposed when the third leaf is emerging, and then the ion analyses on this leaf, which develops into a fully expanded leaf during the salt treatment, are performed after a fixed period of time (e.g. 10 days). In Arabidopsis, the youngest, fully expanded leaf is best for Na and K analyses during stress treatment. We recommend use of a specific leaf for ion analysis to reflect the accumulation of ions in the shoot during the stress. Leaves with visible senescence should not be used because processes controlling ion accumulation are likely to be compromised. As well, the water content of a senescent leaf is also lower than it would be when all the tissues are intact. It should be noted that when collecting root tissue for ion analyses, the roots should be washed briefly (three seconds) in an excess volume of 10 mM CaCl_2_ or 10 mM CaSO_4_ to remove excess nutrient or stress solution (containing high [NaCl]) and to wash away any Na^+^ that adheres to the outside of the root. The Ca^2+^ both displaces Na^+^ from fixed negative charges in the cell walls and stabilizes cell membranes to slow leakages from inside cells. In some cases, such as ion flux measurements, longer rinsing times are necessary (> 10 min). Here it is advisable to adjust the osmotic potential of the rinsing solution (e.g. with sorbitol) to avoid tissue damage of roots with the dilute Ca^2+^ solution (Davenport & Tester, 2000b).

***Section 2: Indices derived from physiological measurements***

***Quantifying the effects of salinity on plant growth***

The salinity tolerance of a plant, in terms of growth, can be expressed using several indices, such as those described below. The equations listed here generally refer to plant biomass, which can be obtained using destructive or non-destructive harvests.

The **relative decrease in plant biomass (RDPB)**, for example, is a simple calculation using the fresh mass of plants (M^­^_f_) at the end of the experiment (time point T_1_), grown under control and salt conditions, such as in the following equation ‎(a):

1. $RDPB=\frac{M_{f, control} - M_{f, salt}}{M_{f, control}}$,

where M_f, control_ is the plant’s fresh mass under control conditions and M_f, salt_ is the plant’s fresh mass under salt conditions.

Using image acquisition techniques, the **shoot ion-independent tolerance index** can be estimated based on the reduction in shoot growth in the first days after salt imposition using equation ‎(b) (Berger *et al.*, 2012). In the classic literature, plant growth can be approximated using an exponential curve, at least over short time periods, and the **relative growth rate (RGR)** is the exponent of this curve. In the calculation of the shoot ion-independent tolerance index, it is crucial to determine RGR in the early stress phase in the first few days after stress imposition before sodium ions have accumulated to toxic levels in the shoot and the sodium has had time to affect shoot metabolism.

1. $Shoot Ionindependent tolerance index= \frac{{RGR}_{salt}}{{RGR}_{control}}$

Five indices have been proposed to describe plants that are more or less tolerant than others. The **salt tolerance (ST)** index is commonly used to compare different accessions and to identify correlations with other traits hypothesized to contribute to salinity tolerance (e.g. Genc *et al.* (2007) correlated sodium content in the shoot with ST). ST can be calculated as in equation ‎(c) using only the endpoint measurement (T_2_). The preferred formula focuses on the interval between T_2_ and T_1_, when stress has been imposed on the plant, as described in ‎(d). The **TOL index** ‎(e) is the simple difference between the trait measured in control and stress conditions (Rosielle & Hamblin, 1981). Selection based on the TOL index favors genotypes with higher yield under stress conditions (compared to control). The **Stress Tolerance Index (STI)** ‎(f) was proposed for yield-related traits. However, it can be adapted for many growth-related traits (Fernandez, 1992). It accounts for both the overall performance of the plant population (e.g. all accessions) under control conditions as well as the ability to maintain yield (or other growth parameters) under stress conditions. A higher Stress Tolerance Index for a genotype indicates a higher stress tolerance potential and a higher yield/growth potential.

The variable Y in these equations denotes growth related traits such as RGR, plant height, total dry mass or even yield. Y_av_ refers to the average of all the genotypes under control conditions for trait Y.

1. $ST= \frac{Y_{salt at T_{2}}}{Y_{control at T_{2}}}$
2. $ST= \frac{Y_{salt at T_{2}} -Y_{salt at T_{1}}}{Y_{control at T_{2}} - Y_{control at T_{1}}}$
3. $TOL index= Y_{control}- Y_{salt}$
4. $Stress Tolerance Index= \frac{Y_{control}}{Y_{av}} \times\frac{Y_{salt}}{Y_{av}}$

The most recognizable indication of salinity stress is a reduction in shoot growth, which in turn changes the allocation of biomass between roots and shoots. This change in allocation of resources can be described using the **root mass ratio (RMR)**. The RMR of a plant grown under control ‎(g) and salt stress ‎(g) conditions can be determined, which allows calculation of the **relative root mass ratio (RRMR)** ‎(i). A plant that has a lower RRMR is reducing the allocation of biomass to the roots to a greater extent than another plant upon salt stress. As described above for ST in equation (d), one should include measurements before and after stress application (at T_1_ and T_2_).

1. ${RMR}_{control}=\frac{M_{d, root,control}}{M_{d, control}}$
2. ${RMR}_{salt}=\frac{M_{d, root,salt}}{M_{d,salt}}$
3. $RRMR =\frac{{RMR}_{salt}}{{RMR}_{control}}$,

where M_d,root,control_ and M_d,root,salt_ are the root’s dry mass under control and salt conditions, respectively, and M_d,control_ and M_d,salt_ are the total plant’s dry mass under control and salt conditions, respectively.

Leaf area generally decreases under saline conditions (Munns & Tester, 2008). The **leaf area ratio (LAR)** is the fraction of the leaf area and its dry mass. The LAR of a plant grown under control ‎(j) and salt stress ‎(k) conditions can be determined, allowing calculation of the **relative leaf area ratio (RLAR)** ‎(l). It can be hypothesized that a decrease in RLAR (i.e. the leaves get thicker in response to salinity) may contribute to salinity tolerance by providing a greater volume of tissue into which salt can be sequestered. Values for the leaf area can be derived from imaging data (for instance using a scanner or digital camera). Again, one should include measurements before and after stress application (at T_1_ and T_2_).

1. ${LAR}_{control}= \frac{A_{leaf,control}}{M_{d,leaf, control}}$

1. ${LAR}_{salt}= \frac{A_{leaf,salt}}{M_{d,leaf,salt}}$
2. $RLAR=\frac{{LAR}_{salt}}{{LAR}_{control}},$

where A_leaf, control_ and A_leaf, salt_ are the leaf’s area under control and salt conditions, respectively. M_d,leaf,control_ and M_d,leaf,salt_ are the leaf’s dry mass under control and salt stress conditions, respectively.

***Quantifying the effects of salinity on a plant’s water relations, transpiration (T) and transpiration use efficiency (TUE)***

Methods to measure classic water relations parameters such as water potential and hydraulic conductivity, are discussed in the main text (section “Quantifying the effects of salinity on plant water relations, transpiration (T) and transpiration use efficiency (TUE)”), where the reader is referred to the extensive classical plant physiological literature in this area.

The **water fraction (WF)** of a tissue can be calculated using the fresh and dry mass of the tissues under control ‎(m) or saline conditions ‎‎(n). The **relative water fraction (RWF)** described in ‎‎(o) indicates how much the water fraction is reduced during salinity treatment, as the ratio between the two parameters, WF_salinity_ and WF_control_. For instance, a plant that has a higher RWF value is able to maintain its water content in the shoot to a greater extent than a plant with a lower RWF upon salt stress.

1. ${WF}_{control}= \frac{M_{f,shoot,control} - M_{d,shoot,control}}{M_{f,shoot,control}}$

1. ${WF}_{salt}= \frac{M_{f,shoot,salt} - M_{d,shoot,salt}}{M_{f,shoot,salt}}$
2. $RWF= \frac{{WF}_{salt}}{{WF}_{control}},$

where M_f,shoot,control_ and M_f,shoot,salt_ are the shoot’s fresh mass under control and salt conditions, respectively. M_d,shoot,control_ and M_d,shoot,salt_ are the shoot’s dry mass under control and salt conditions, respectively.

Relative water content (RWC) ‎(p) has been extensively used in the classical literature to determine the water status of a shoot relative to its fully hydrated state. To measure RWC, leaves are detached and floated for long periods on deionized water, potentially causing over-hydrating of leaves, with liquid in intercellular spaces, as well as cellular damage. This problem can be amplified in leaves from salt-treated plants that have undergone osmotic adjustment, as even more water will move into the tissue because of its more negative osmotic potential. However, with careful procedures, relative water content can be measured accurately. Rehydration should be done by placing just a cut petiole into a rehydration solution; leaves should not be simply floated on a solution (Sade *et al.*, 2015). Rehydrating with a dilute CaCl_2_ solution helps maintain cell integrity. Observing the leaf microscopically after rehydration for intercellular water allows over-rehydration, which would lead to artificially low values for RWC values (Boyer *et al.*, 2008), to be monitored. From measures of leaf mass upon excision, leaf mass after careful rehydration and dry leaf mass, RWC can be calculated as:

1. $RWC= \frac{M_{f} - M_{d}}{M_{t}- M_{d}},$

where M_t_ is the mass of the leaf after rehydration (the “turgid mass”) and the other symbols are as used previously. RWC can then be measured in control and salt-treated plants, and the effect of salinity on RWC can be calculated in a similar way to that described for other traits above.

If preferred, relative water deficit can also be determined simply from 1-RWC. This can give numbers that are much smaller (in the order of 0.02 to 0.04, for example, rather than 0.96 to 0.98) so that relative changes appear to be larger).

To integrate measures of transpiration over space and time and to overcome the need for complex instruments and formulae to estimate transpiration, pots containing plants can be weighed at frequent intervals and transpiration can be derived from the decrease in pot mass. Water loss occurs by transpiration from the plant and evaporation from the soil surface; the latter must be minimized and measured separately to determine the transpiration of the plant accurately. The effects of salinity can be measured simply by comparing transpiration rates in control and salt-treated plants. By also quantifying plant biomass through the experiment using non-destructive imaging, **transpiration use efficiency (TUE)** at the whole-plant level can be calculated across time, as in equation ‎(q):

1. $TUE= \frac{M_{total}}{M_{water transpired}}$,

where M_total_ is the biomass produced and M_water transpired_ is the amount of water transpired by the whole plant.

***Quantifying the effects of salinity on plant senescence***

Processes that involve premature senescence, particularly of older leaves, can be recorded using visual scores (e.g. Table 2) or imaging. Plants can be assessed after they have been exposed to salinity for an extended period, when clear symptoms of salt toxicity are visible. The imaging can be done either by scanning the leaves manually (e.g. in a commercial scanner) or in an automated set-up using, for instance, high-throughput fluorescent imaging. This imaging analysis allows calculation of the area affected by **salt-induced senescence (SIS)** (Rajendran *et al.*, 2009; Berger *et al.*, 2012) ‎(r), calculated as:

1. $SIS=\frac{A_{senesc,salt}}{A_{shoot,salt}}- \frac{A_{senesc,control}}{A_{shoot,control}}$ ,

where A_senesc,salt_ and A_senes,control_ are the area that is senescing under salt and control conditions, respectively. A_shoot,salt_ and A_shoot,control_ are the projected shoot area under salt and control conditions, respectively.

In statistical analyses, the modes or medians of visual scores are the most suitable way to interpret the results. The mode or median is preferable to the mean/average, since the observed values are ordinal data (i.e. numbers allocated to rankings, not a quantitative value). Generally, the display of continuous data is preferable as box plots, scatter-plots, histograms or in some cases line graphs (e.g. for dose-response curves) as these allow the reader to evaluate data and its distribution (Weissgerber *et al.*, 2015).

***Quantifying the effects of salinity on yield-related parameters***

**Harvest index (HI)**, as described in equation ‎(s), indicates the grain or fruit produced per unit of total above-ground biomass of the plant. Salinity stress decreases shoot growth; however, if a plant is able to maintain grain or fruit production under stress, HI will be greater compared to that under control conditions.

1. $HI= \frac{M_{grain/fruit}}{M_{A}},$

where M_grain/fruit_ is the biomass of the grain or fruit and M_A_ is the above-ground biomass.

For example, in monocotyledons, some of the measurable yield components are tillers per plant, ears per tiller, ears per m^2^, grains per ear, mass per grain, and grain mass per plant. Some of these components are strongly affected by planting density, fertilizer application and/or watering, constituting a potential source of variation. In an experimental setting, it is particularly important to keep the variation that results from field management to a minimum. Verhulst *et al.* (2013) compiled a detailed handbook on how to determine yield and yield components of grain crops.

**Armengaud P, Zambaux K, Hills A, Sulpice R, Pattison RJ, Blatt MR, Amtmann A. 2009.** EZ-Rhizo: integrated software for the fast and accurate measurement of root system architecture. *The Plant Journal* **57**(5): 945-956.

**Berger B, de Regt B, Tester M. 2012.** Trait dissection of salinity tolerance with plant phenomics. *Methods in Molecular Biology (Clifton, N.J.)* **913**: 399-413.

**Black J. 1956.** The influence of seed size and depth of sowing on pre-emergence and early vegetative growth of subterranean clover *Trifolium subterraneum* L. *Australian Journal of Agricultural Research* **7**(2): 98-109.

**Boyer JS, James RA, Munns R, Condon TAG, Passioura JB. 2008.** Osmotic adjustment leads to anomalously low estimates of relative water content in wheat and barley. *Functional Plant Biology* **35**(11): 1172-1182.

**Boyes DC, Zayed AM, Ascenzi R, McCaskill AJ, Hoffman NE, Davis KR, Görlach J. 2001.** Growth stage–based phenotypic analysis of Arabidopsis: a model for high throughput functional genomics in plants. *The Plant Cell* **13**(7): 1499-1510.

**Conn SJ, Hocking B, Dayod M, Xu B, Athman A, Henderson S, Aukett L, Conn V, Shearer MK, Fuentes S, et al. 2013.** Protocol: optimising hydroponic growth systems for nutritional and physiological analysis of *Arabidopsis thaliana* and other plants. *Plant Methods* **9**(1): 4.

**Corwin DL, Lesch SM. 2013.** Protocols and guidelines for field-scale measurement of soil salinity distribution with ECa-directed soil sampling. *Journal of Environmental & Engineering Geophysics* **18**(1): 1-25.

**Cramer GR, Lauchli A, Polito VS. 1985.** Displacement of Ca^2+^ by Na^+^ from the plasmalemma of root cells- a primary response to salt stress. *Plant Physiology* **79**(1): 207-211.

**Cramer GR, Lynch J, Lauchli A, Epstein E. 1987.** Influx of Na^+^, K^+^, and Ca^2+^ into roots of salt-stressed cotton seedlings - effects of supplemental Ca^2+^ *Plant Physiology* **83**(3): 510-516.

**Davenport RJ, Tester M. 2000a.** A weakly voltage-dependent, nonselective cation channel mediates toxic sodium influx in wheat. *Plant Physiology* **122**(3): 823-834.

**Davenport RJ, Tester M. 2000b.** A weakly voltage-dependent, nonselective cation channel mediates toxic sodium influx in wheat. *Plant Physiology* **122**(3): 823-834.

**Duan L, Dietrich D, Ng CH, Chan PMY, Bhalerao R, Bennett MJ, Dinneny JR. 2013.** Endodermal ABA signaling promotes lateral root quiescence during salt stress in Arabidopsis seedlings. *The Plant Cell Online*.

**Epstein E. 1961.** Essential role of calcium in selective cation transport by plant cells. *Plant Physiology* **36**(4): 437-&.

**Fernandez GCJ 1992**. Effective selection criteria for assessing plant stress tolerance. *Proceedings of the international symposium on adaptation of vegetable and other food crops in temperature and water stress*. Taiwan. pp. 257-270.

**Genc Y, McDonald GK, Tester M. 2007.** Reassessment of tissue Na^+^ concentration as a criterion for salinity tolerance in bread wheat. *Plant Cell and Environment* **30**(11): 1486-1498.

**Genc Y, Tester M, McDonald GK. 2010.** Calcium requirement of wheat in saline and non-saline conditions. *Plant and Soil* **327**(1-2): 331-345.

**Graves WR. 1992.** Influence of hydroponic culture method on morphology and hydraulic conductivity of roots of honey locust. *Tree Physiology* **11**(2): 205-211.

**Gregorio GB, Senadhira D, Mendoza RD 1997**. Screening rice for salinity tolerance. *IRRI discussion papers No. 22*. Manila (Philippines): International Rice Research Institute.

**Jha D, Shirley N, Tester M, Roy SJ. 2010.** Variation in salinity tolerance and shoot sodium accumulation in Arabidopsis ecotypes linked to differences in the natural expression levels of transporters involved in sodium transport. *Plant, Cell & Environment* **33**(5): 793-804.

**Julkowska MM, Hoefsloot HCJ, Mol S, Feron R, de Boer GJ, Haring MA, Testerink C. 2014.** Capturing Arabidopsis root architecture dynamics with ROOT-FIT reveals diversity in responses to salinity. *Plant Physiology* **166**(3): 1387-1402.

**Lahaye PA, Epstein E. 1969.** Salt toleration by plants: enhancement with calcium. *Science* **166**(3903): 395-396.

**Maas EV, Hoffman GJ. 1977.** Crop salt tolerance - current assessment. *Journal of the Irrigation and Drainage Division, American Society of Civil Engineers* **103**(IR2): 115-134.

**Malik AI, English JP, Colmer TD. 2009.** Tolerance of *Hordeum marinum* accessions to O_2_ deficiency, salinity and these stresses combined. *Annals of Botany* **103**(2): 237-248.

**Munns R, James RA. 2003.** Screening methods for salinity tolerance: a case study with tetraploid wheat. *Plant and Soil* **253**(1): 201-218.

**Munns R, Tester M. 2008.** Mechanisms of salinity tolerance. *Annual Review of Plant Biology* **59**: 651-681.

**Poorter H, Bühler J, van Dusschoten D, Climent J, Postma JA. 2012.** Pot size matters: a meta-analysis of the effects of rooting volume on plant growth. *Functional Plant Biology* **39**(11): 839-850.

**Poorter H, Fiorani F, Stitt M, Schurr U, Finck A, Gibon Y, Usadel B, Munns R, Atkin OK, Tardieu F, et al. 2012.** The art of growing plants for experimental purposes: a practical guide for the plant biologist Review. *Functional Plant Biology* **39**(10-11): 821-838.

**Rajendran K, Tester M, Roy SJ. 2009.** Quantifying the three main components of salinity tolerance in cereals. *Plant Cell and Environment* **32**(3): 237-249.

**Rengasamy P. 2006.** World salinization with emphasis on Australia. *J Exp Bot* **57**(5): 1017-1023.

**Rhoades JD, Chanduvi F, Lesch SM 1999.** Salinity Asessment: Methods and Interpretation of Electrical Conductivity Measurements. *Procedure for interpreting soil salinity*. Food and Agriculture Organization, Rome.

**Rosielle AA, Hamblin J. 1981.** Theoretical aspects of selection for yield in stress and non-stress environments. *Crop Science* **21**(6): 943-946.

**Sade N, Shatil-Cohen A, Moshelion M. 2015.** Bundle-sheath aquaporins play a role in controlling Arabidopsis leaf hydraulic conductivity. *Plant Signaling & Behavior* **10**(5): e1017177.

**Schaff J, Schultz B, Craft E, Clark R, Kochian L. 2010.** Geochem-EZ: a chemical speciation program with greater power and flexibility. *Plant and Soil* **330**: 207-214.

**Sharma PC, Kumar P. 1999.** Alleviation of salinity stress during germination in *Brassica juncea* by pre-sowing chilling treatments to seeds. *Biologia Plantarum* **42**(3): 451-455.

**Shavrukov Y. 2013.** Salt stress or salt shock: which genes are we studying? *Journal of Experimental Botany* **64**(1): 119-127.

**Shavrukov Y, Genc Y, Hayes J 2012.** The use of hydroponics in abiotic stress tolerance research. In: Asao T ed. *Hydroponics - a standard methodology for plant biological researches*: InTech.

**Singh RK, Gregorio GB, Ismail AM. 2008.** Breeding rice varieties with tolerance to salt stress. *Journal of Indian Society of Coastal Agricultural Research* **26**: 16-21.

**Taeb M, Koebner RMD, Forster BP, Law CN. 1992.** Association between genes-controlling flowering time and shoot sodium accumulation in the Triticeae. *Plant and Soil* **146**(1-2): 117-121.

**Tavakkoli E, Fatehi F, Rengasamy P, McDonald GK. 2012.** A comparison of hydroponic and soil-based screening methods to identify salt tolerance in the field in barley. *Journal of Experimental Botany* **63**(10): 3853-3867.

**Tavakkoli E, Rengasamy P, McDonald GK. 2010.** The response of barley to salinity stress differs between hydroponic and soil systems. *Functional Plant Biology* **37**(7): 621-633.

**Tester M, Davenport R. 2003.** Na^+^ tolerance and Na^+^ transport in higher plants. *Annals of Botany* **91**(5): 503-527.

**Verhulst N, Cox R, Govaerts B. 2013.** *Yield and yield components: A practical guide for comparing crop management practices*: Mexico, DF (Mexico) : CIMMYT. <http://repository.cimmyt.org/>.

**Volkov V, Wang B, Dominy PJ, Fricke W, Amtmann A. 2004.** *Thellungiella halophila*, a salt-tolerant relative of *Arabidopsis thaliana*, possesses effective mechanisms to discriminate between potassium and sodium. *Plant Cell and Environment* **27**(1): 1-14.

**Weissgerber TL, Milic NM, Winham SJ, Garovic VD. 2015.** Beyond bar and line graphs: time for a new data presentation paradigm. *PLoS Biology* **13**(4): e1002128.

**Wetson AM, Flowers TJ. 2010.** The effect of saline hypoxia on growth and ion uptake in *Suaeda maritima*. *Functional Plant Biology* **37**(7): 646-655.

**Supplementary Figure 1** Sensitivity of rice to salt varies during the life cycle (image from (Singh *et al.*, 2008), with the author’s permission.

**Supplementary Table 1** Range of NaCl concentrations for use with different species in hydroponics, soil-filled pots and field experiments.

|  | **Advisable salinity levels for hydroponics (mM NaCl)** | **Salinity levels in pot experiments (mM NaCl)** | **Threshold for yield losses in field experiments (EC_e_ in dS/m) *** |
| --- | --- | --- | --- |
| **Arabidopsis** | 75-100 | 100-150 | N/A |
| **Rice** | 50-75 | 80-150 | 3-4 |
| **Barley** | 150-200 | 200-250 | 8-10 |
| **Bread wheat** | 100-150 | 150-200 | 6-8 |
| **Tomato** | 100-150 | 100-200 | 2.5 |

Note: Values stated are recommendations and will vary according to genotype, plant age and environmental factors (such as relative humidity).

* Values for field experiments are based on Maas and Hoffman (1977) and taken from <http://www.fao.org/docrep/005/y4263e/y4263e0e.htm>. EC_e_ refers to the electrical conductivity of the saturated paste.
