# Supplementary figures and images for: Evaluating physiological responses of plants to salinity stress
Source: Ann Bot. 2016 Sep 5;119(1):1–11. doi: 10.1093/aob/mcw191 (PMC5218372; doi:10.1093/aob/mcw191)

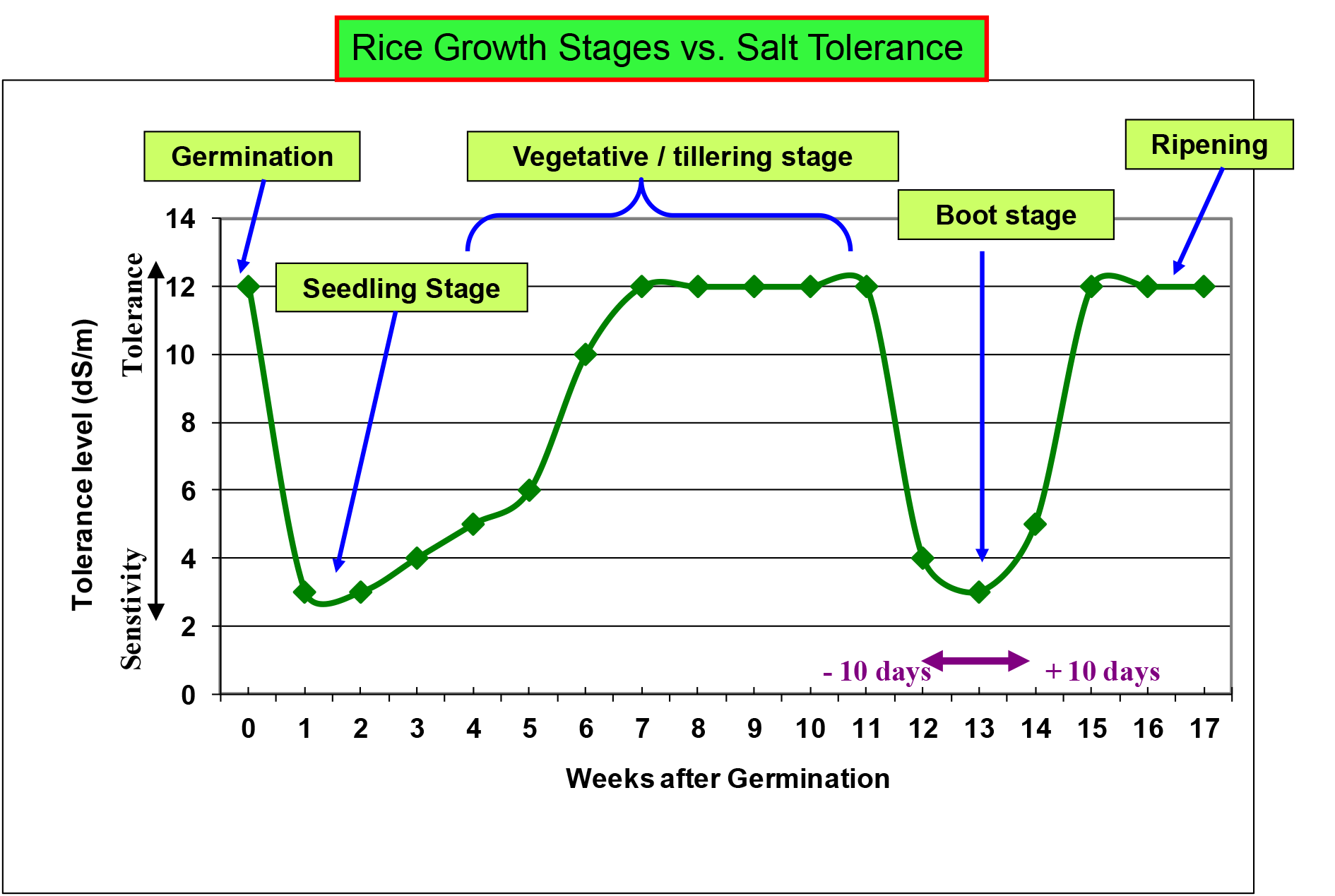

Supplement: Supplementary Data [file supp_mcw191_suppl_data.zip › aob-16508-s03.tif]
